# Design and implementation of a climate chamber for moisture sensitive nanotomography of biological samples

**DOI:** 10.1107/S1600577525006484

**Published:** 2025-08-18

**Authors:** Martin Nopens, Imke Greving, Silja Flenner, Linnea Hesse, Jan Lüdtke, Michael Altgen, Gerald Koch, Johannes Beruda, Sabrina Heldner, Hannes Köhm, Sergej Kaschuro, Andrea Olbrich, Jakob Benedikt Mietner, Fabian Scheckenbach, Jördis Sieburg-Rockel, Andreas Krause

**Affiliations:** a Thünen Institute of Wood Research, Leuschnerstraße 91c, 21031Hamburg, Germany; b Institute of Materials Physics, Helmholtz-Zentrum Hereon, Max-Planck-Str. 1, 21502Geesthacht, Germany; c Institute of Wood Science, Biomimetics Universität Hamburg E Ohnhorststraße 18 22609Hamburg Germany; d Cluster of Excellence livMatS @ FIT – Freiburg Center for Interactive Materials and Bioinspired Technologies, University of Freiburg, 79110Freiburg im Breisgau, Germany; e Norwegian Institute of Bioeconomy Research Høgskoleveien 8 1433Ås Norway; Bhabha Atomic Research Centre, India

**Keywords:** *in situ*, nanotomography, climate chamber, relative humidity, biological specimen

## Abstract

Deep understanding of the structural composition and growth of biological specimens is becoming increasingly important for the development of bio-based and sustainable material systems. Full-field nano-computed tomography is particularly suitable for this purpose as it allows for non-destructive 3D imaging at high spatial resolution. However, most biological samples are functionalized by water and respond sensitively to any changes in climate conditions, specifically relative humidity, by adjusting their material moisture content. To date, only a limited number of tomography instruments offer an *in situ* climate control option to users. These, however, are limited either by the range of relative humidity states, the long times required to change the climate state, or obstruction or attenuation of the beam. Here, the first fully automatized climate cell for *in situ* full-field nanotomography is presented. It has been designed, built and integrated at the nanotomography station at the P05 imaging beamline, operated by Hereon at the DESY storage ring PETRA III, Germany. The highly flexible and windowless design allows the humidity dependent swelling and shrinking of lignified plant cell walls to be studied *in situ*, using phase contrast nanotomography. The concept of this climate chamber can easily be integrated into other setups. It operates in the relative humidity range of 0–90% and can be controlled in a temperature range of 10–50°C. Climate conditions can be adjusted at any time, remotely from the control hutch by using a humidity generator. Results show that the developed setup maintains a stable climate during the entire duration of a tomographic scan at different humidities and does not obstruct the sample or hinder the imaging conditions. During the tomographic investigation the sample remains stable in the flow of the air stream and shows typical cell wall swelling and shrinking behaviour depending on the equilibrium moisture content. This new climate cell is now available to all users of the P05 nanotomography instrument for conditioning samples, serving a wide range of scientific applications.

## Abstract 2

Design and implementation of an *in situ* climate chamber for nanotomography allowing the analysis of moisture sensitive specimens at different relative humidity and temperature settings at the nanotomography imaging station of beamline P05 (operated by Hereon, at the PETRA III storage ring, DESY, Germany).

## 1. Introduction

In order to optimize and expand the use of lignocellulose-based material systems, we need to further analyse the relationships between material, structure and function across multiple levels of structural hierarchy, *e.g.* for the taxonomic identification of wood specimens (Dierickx *et al.*, 2024 ▸). So far, knowledge about the nanoscopic level, the level of material–water interaction causing the swelling and shrinkage of wood, is limited. Nanotomography is the only non-destructive imaging technique available to investigate the 3D internal structure of organic materials *in situ* at relevant time scales, in various climate conditions, and at the required spatial resolution.

In general, biological samples are influenced by and respond to external conditions such as temperature, light, humidity, *etc*. In plants, water can lead to changes in material properties (stiffness changes) and structure (deformation, swelling and shrinking) but can also trigger different active and/or passive processes such as movement, growth, transport *etc*. (Derome *et al.*, 2012 ▸; Dumais & Forterre, 2012 ▸). Therefore, stable climate conditions during the whole experiment are of crucial importance to avoid artefacts and obtain high quality results in X-ray nanotomography.

For this purpose, climate chambers for *in situ* analysis have been developed for different large-scale facility based techniques, *e.g.* in diffraction and scattering experiments (Esmaeili *et al.*, 2013 ▸; Jackson *et al.*, 2013 ▸; La Salas-de Cruz *et al.*, 2012 ▸; Katsaras & Watson, 2000 ▸; Kim *et al.*, 2005 ▸; Case *et al.*, 2017 ▸). Suitable setups where the climate can be controlled have also been developed for scanning X-ray tomographic experiments. For instance, an air handling and conditioning system was used to mix dry and saturated water vapour in a climate system at TOMCAT (Derome *et al.*, 2011 ▸), with the requirement of membrane sealing to close the gap between climate chamber and rotational stage. Samples were investigated at different relative humidity (r.h.) within plastic tubes at ESRF ID19 (Gamstedt *et al.*, 2015 ▸; Joffre *et al.*, 2016 ▸), which requires the samples to be removed from the setup for changing climate conditions. A humidity generator was used at different r.h. for climatizing samples at the Advanced Photon Source beamline 2-BM-B at Argonne National Laboratory (Arzola-Villegas *et al.*, 2023 ▸). A climate chamber designed for high temperatures for ptychographic tomograms (scanning method) was developed at the flOMNI microscope at the Swiss Light Source (Holler *et al.*, 2022 ▸). Recently the option of measuring at different relative humidity levels was added (Gao *et al.*, 2024 ▸). Often these climate cells have the holes for the beam path covered with Kapton or polyamide foil as window material (Katsaras & Watson, 2000 ▸; Derome *et al.*, 2011 ▸). Classical laboratory computed tomographic setups also offer experiments across a wide humidity range (Vonk *et al.*, 2019 ▸), where a dedicated cell had to be designed. However, major challenges need to be overcome when it comes to high spatial resolution full-field nano-imaging at a synchrotron source. Here, the nature of the imaging geometry and the requirements on sample stability do add an extra layer of complexity to the design of such a climate cell. To the best of our knowledge, no full-field nano-computed tomography device currently exists which combines a 180° rotatable and windowless climate cell with tunability of relative humidity and temperature that was specifically designed for biological materials.

Apart from the fact that *in situ* devices for tomography should ideally not obstruct the X-ray beam during the 180° rotation, other equally important points need to be considered in the design of such a climate cell. For high-resolution applications, like full-field nanotomography, a specific focus has to be on the setup stability, such as, for example, avoiding any type of long-term drift or vibrational motion in order to minimize image artefacts. Therefore, any mechanical connection between the rotation axis and the climate cell should be avoided, since it is a possible source of vibration or other disturbance causing image artefacts. On top of that, the climate conditions (*i.e.* temperature and r.h.) must of course be tunable in a precise way, and the climate must be very stable (±1–3%) during a typical tomographic scan time (>30 min). Another challenge arises when using phase contrast methods, *e.g.* near-field holotomography (Flenner *et al.*, 2020*a* ▸). Here any kind of window material, obstructing the beam path, is prone to inducing artefacts in the phase reconstruction and has therefore also to be avoided. These specific challenges have not been met for any full-field nanotomography beamline setup, to the best of our knowledge.

With the aim of studying wood and other lignified samples at defined climate conditions at sub-micrometre resolution in 3D a suitable cell was designed. The ultimate aim was to develop an *in situ* climate chamber that allowed for a wide and adjustable relative humidity and temperature range, a stable climate during image acquisition, a fast and easy sample exchange, and a 180° sample rotation without beam blockage in order to minimize artefacts induced by limited angle tomography. Furthermore, the climate chamber should avoid inducing vibrations, and it should be applicable to different modes of operation offered at the P05 nanotomography beamline, *e.g.* methods such as transmission X-ray microscopy (TXM) and near-field holotomography (NFHT). The diameter of such a climate cell was chosen to be as small as possible to allow for small working distance optics. In addition, the presented design needed to be windowless. This is not only to reduce beam attenuation but more importantly to minimize reconstruction artefacts resulting from particle deposition on the window material (caused, for example, by X-ray irradiation or electrostatic forces). Finally, the technical setup should be designed such that the sample is stable despite the in-stream of conditioned air.

## 2. Material and methods

### 2.1. Climate chamber

**Figure 1:**
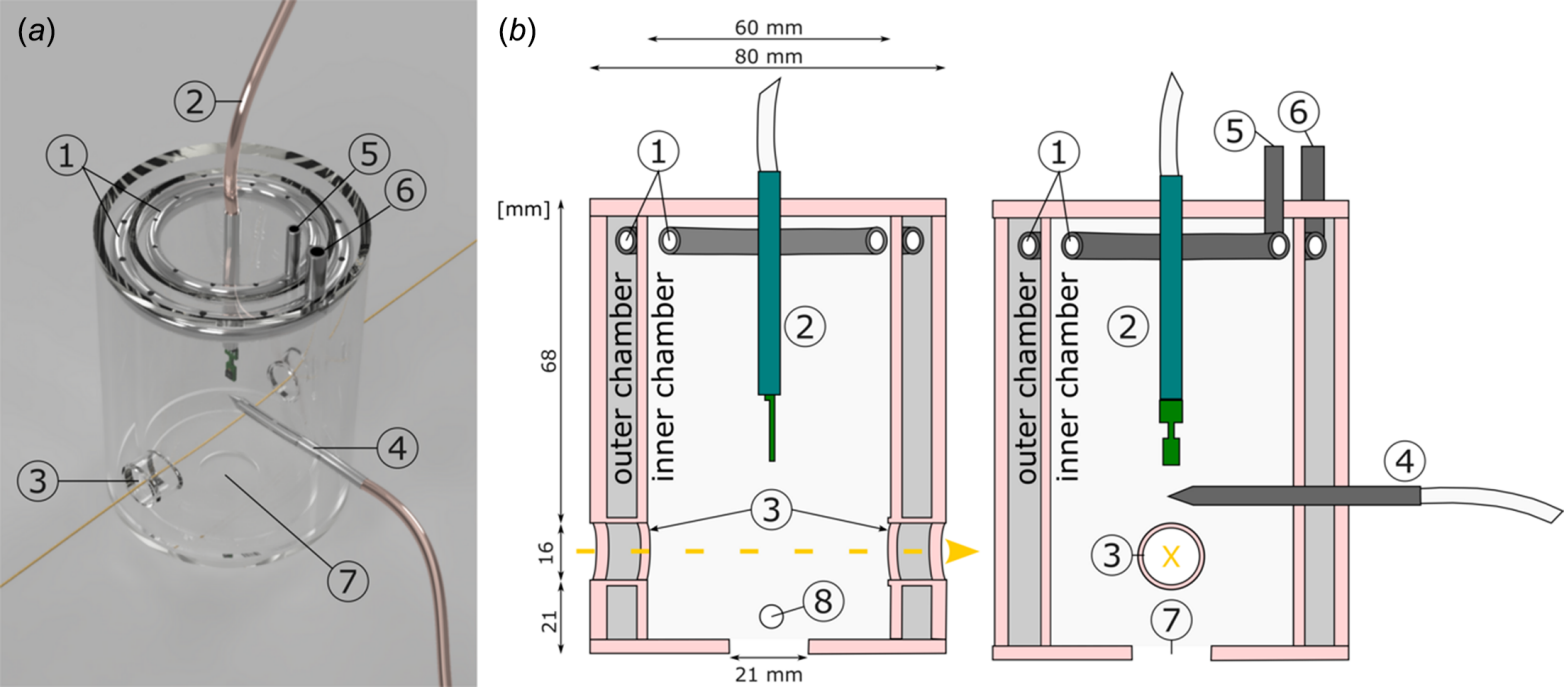
Photograph (*a*) and schematic drawing (*b*) of two vertical sections of the climate chamber. Two polymethyl methacrylate pipes (pink; respective outer diameters of 80 mm and 60 mm) are attached to two discs (pink top and bottom) using UV adhesive. The metal ring jets (1) are connected to an outlet for relative humidity (5; ring jet in inner chamber) and cooling and heating (6; ring jet in outer chamber), respectively. The inner chamber is equipped with an Arduino sensor (2) and a modular humidity generator (MHG) sensor (4). Two opposite holes in the chamber (3) allow for an unobstructed passage of X-rays (yellow arrow/X). A bottom opening (7) and a side opening in the outer chamber (8) allow conditioned air to exit.

A climate chamber (Fig. 1 ▸) was designed and commissioned for the nanotomography branch of the imaging beamline P05, operated by the Helmholtz-Zentrum Hereon at the PETRA III storage ring at DESY. The flexibility of the setup allows for TXM including Zernike phase contrast (ZPC) and NFHT, which can be performed in just a few minutes (Flenner *et al.*, 2020*a* ▸; Longo *et al.*, 2020 ▸).

The climate chamber consists of two polymethyl methacrylate pipes (Fig. 1 ▸; diameters 80 mm and 60 mm), the smaller pipe inserted into the larger pipe, which are glued onto two discs (base and top of climate chamber) using UV adhesive. This allows the chamber to be positioned over the rotation stage without touching it.

The double-chamber design facilitates independent temperature regulation, which is critical for preventing condensation under conditions of low temperature and high relative humidity; for example, by pre-cooling the chamber prior to the introduction of humidified air (<60% r.h.). This precaution is essential because the modular humidity generator [MHG; see (2) in Fig. 1 ▸] is not able to equilibrate with the ambient temperature of the chamber, *i.e.* it can only control the temperature of its air output. Consequently, if humidified air is let into an unconditioned chamber environment, the resulting open thermodynamic system may lead to condensation due to local temperature differences.

If condensation occurs, it can affect supply lines, the chamber and, in the worst case, the sample and its holder. Therefore, pre-conditioning the chamber and sample to a specific temperature is essential. Since MHG values are instantly recorded and displayed at the control panel, users can easily detect the onset of condensation through unstable or elevated relative humidity values. Dry airflow can then be injected to prevent further condensation.

An inner and an outer ring jet with punctiform openings as air outlets were handcrafted from metal. A Parker Origa Pneumatic linear axis drive OSP-P is used for pneumatically lifting the climate chamber vertically. The remote controllable automatic pneumatic system enables a quick and user-friendly sample change, while keeping the climate chamber at a permanent distance of 1–2 mm above the rotation stage.

**Figure 2:**
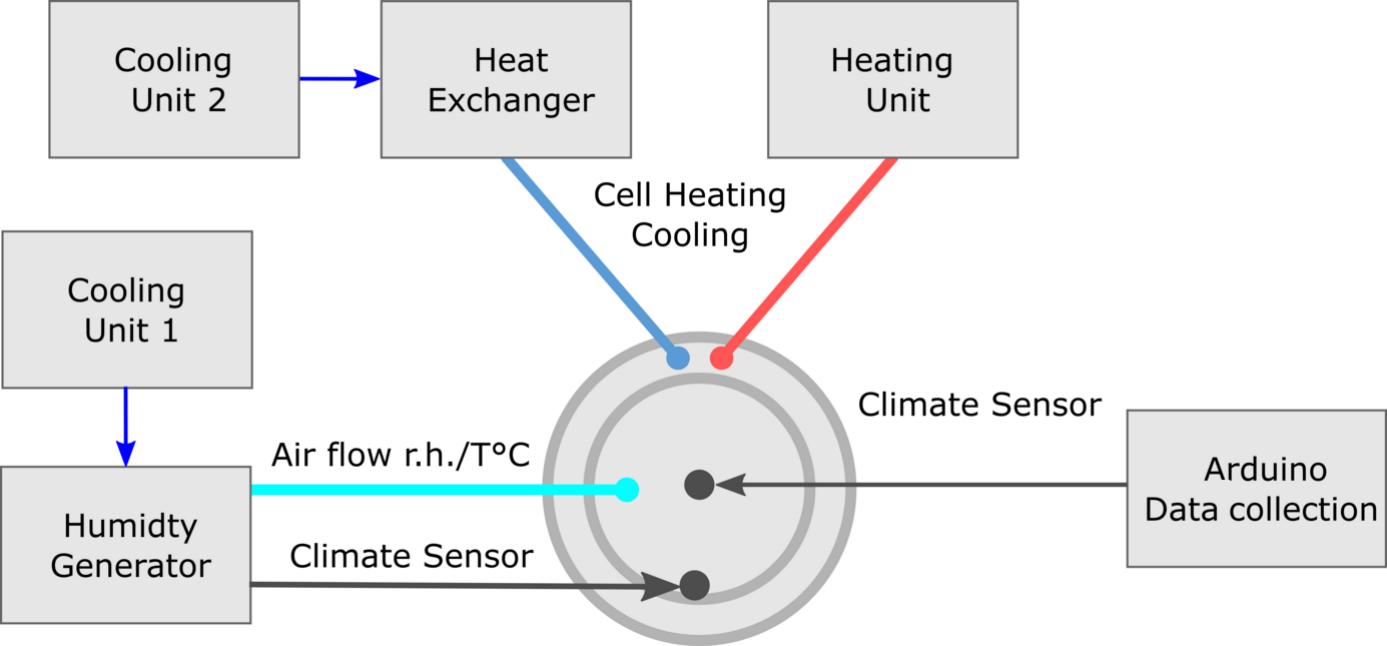
Schematic representation of the supply lines of the climate chamber with all used devices and relevant interconnections.

The temperature in the climate chamber is controlled by adjusting the airflow temperature in the air space between the two polymethyl methacrylate cylinders (outer chamber in Fig. 1 ▸) as the outer ring jet floods cool or warm air into the space [(1) and (6) in Fig. 1 ▸]. Heating is achieved via an LE Mini Sensor Kit air heater, 230 V/800 W (Leister Technologies Deutschland GmbH, Germany), while cooling of the air space in the outer chamber as well as cooling of the humidity generator is realized by an IKA RC 2 basic cooling unit (IKA-Werke GmbH, Germany). Heat exchange is enabled using an EWT-B3-12 heat exchanger (from LUPI Präzisionstechnik GmbH, Germany). Supply lines consist of encased silicone hoses. An overview of all connections is shown in Fig. 2 ▸.

Conditioned air from the sample chamber [inner chamber in Fig. 1 ▸(*b*)] can exit the climate chamber through the openings for the beam path [(3) in Fig. 1 ▸] or at the bottom opening [(7) in Fig. 1 ▸]. The air for temperature adjustments in the outer chamber [Fig. 1 ▸(*b*)] exits through openings at the lower end of the climate chamber [(8) in Fig. 1 ▸(*b*)].

A modular humidity generator, MHG100 (ProUmid GmbH & Co. KG, Germany), equipped with a Hygroclip HC2-C04 sensor (Rotronic, Germany), with an accuracy of ±0.8% r.h. and ±0.1°C, humidified the air of the inner main chamber at a flow rate of 8 l min^−1^ [(1) and (5) in Fig. 1 ▸; Table 1 ▸]. Dry and oil-free compressed air is used. An Arduino Uno/Mega, Software Version Arduino 1.8.15, is used to validate and control the SHT85 humidity and temperature sensor [(2) in Fig. 1 ▸], (Sensirion, Switzerland) with an accuracy of ±1.5% r.h. and ±0.1°C.

Care was taken in the design of the windowless approach for the beam path through the cell. The openings [entrance and exit window, (3) in Fig. 1 ▸] are implemented in the climate chamber to ensure a non-disturbed or obstructed beam passage through the chamber. This design allows windows to be avoided and therefore avoids artefact induction, as discussed above. The flow of the carrier gas within the chamber has been optimized by smoke-enriched air, tested at different flow rates to ensure stable climate conditions near the sample position without interfering with the beam passage. Thanks to this windowless approach, the X-rays are not attenuated and, more importantly, not disturbed due to particle depositions on any window material. The latter is very important when it comes to NFHT, since it would affect flat-field correction of the projections, causing artefacts in the phase reconstructions.

### 2.2. Experimental setup for nanotomography at beamline P05

The hard X-ray nanotomography setup at the P05 imaging beamline, operated by the Helmholtz-Zentrum Hereon at the PETRA III storage ring at DESY, is specifically designed for *in situ* applications (Flenner *et al.*, 2020 ▸*a*; Flenner *et al.*, 2020 ▸*b*; Longo *et al.*, 2020 ▸). Optics with large focal distances (from 45 mm up to 130 mm) are used to allow for extended sample environments. The energy range for the nanotomographic experiments can be varied in the range 8–17 keV. Imaging of biological specimens at energies above 10 keV has several advantages over soft X-ray nanotomography with respect to dose, penetration depth and depth of focus. The absorption contrast, however, is often very weak. Contrast enhancements through contrast agents often lead to unwanted side effects, particularly when the native state of biological samples is of interest. This issue can be overcome by using phase contrast imaging techniques, such as NFHT or ZPC.

Both methods, NFHT and ZPC, are available at the P05 imaging beamline. The windowless approach of the climate chamber [(3) in Fig. 1 ▸] reduces artefacts in the phase reconstruction of methods like NFHT. The size of the window opening of the cell was designed such that the flat-field images (beam profile without sample) can be acquired by moving the whole cell perpendicular to the beam. Vacuum tubes were installed in the beam path between the climate chamber and the detector to reduce the absorption of X-rays by air. The fast-imaging modalities offered at P05 nanoCT are ideal to study, for example, the swelling mechanism of single plant cells.

### 2.3. Material

Lignified tissue of the endocarp of a *Hura crepitans* fruit was used. Samples were prepared by manually cutting individual cells using a razor blade and positioning them on specialized sample holders for the nanotomography setup using UV adhesive. The sample was first measured at r.h. 90% and then at r.h. 0.9% while the flow rate of the air was set to 8 l min^−1^. The presented exemplary experiment was carried out at room temperature (22.5°C). The feasibility of the method was additionally tested on different wood material (earlywood and latewood) and also at different temperature states (data not shown).

### 2.4. Image acquisition

Images were acquired using the near-field holotomography setup (Flenner *et al.*, 2020*a* ▸) at the P05 imaging beamline operated by Helmholtz-Zentrum Hereon. A gold Fresnel zone plate with a diameter of 300 µm and an outermost zone width of 50 nm was used for focusing the beam. At an X-ray energy of 11 keV, the samples were scanned with an exposure time of 0.8 s using a Hamamatsu sCMOS camera detector (Hamamatsu C12849-101U, 6.5 µm pixel size, 2048 × 2048 pixel, 16-bit image depth) equipped with a 10 µm Gadox scintillator. The detector was placed 19.60 m behind the sample, thus preventing any kind of light optical magnification (Flenner *et al.*, 2020 ▸*b*). The sample was placed at a defocus distance of 181.7 mm. A single defocus distance was sufficient for phase reconstruction, using the iterative algorithm from Hagemann *et al.* (Hagemann *et al.*, 2018 ▸; Dora *et al.*, 2025 ▸) integrated in the *HoloTomoToolbox* (Lohse *et al.*, 2020 ▸). Each tomographic scan took 20 min, acquiring 1500 projection images and 100 references. The tomographic reconstruction was performed using the gridrec algorithm (Dowd *et al.*, 1999 ▸) and a Shepp–Logan filter using *TomoPy* (Gürsoy *et al.*, 2014 ▸).

We checked the influence of different humidity levels in the climate chamber and/or sample on our image quality by taking the average of 100 reference images with dry and humid air and determining the counts/pixel in both states. Comparing the average count rate per pixel (mean) over the entire images showed no significant difference of both states. A value of 5709 ± 8 counts pixel^−1^ (dry state) and 5711 ± 21 counts pixel^−1^ (humid state) were determined showing no influence of the sample condition on image quality.

### 2.5. Image analysis

The changes of relative humidity between r.h. 90% (wet) and r.h. 0.9% (dry) leads to swelling and shrinkage of the cell wall of the scanned samples. The volume change of the cell wall is analysed using Amira-AVIZO 3D (Thermo Fisher Scientific, Waltham, Massachusetts, USA). For this purpose, affine image registration is applied in order to determine the scale factor of the humidity induced cell wall deformation (Patera *et al.*, 2018 ▸*a*; Patera *et al.*, 2018 ▸*b*). For pre-processing, the greyscale images (16-bit) are first converted to 8-bit in order to reduce computational time. Subsequently, the volumes are masked to remove the air before aligning the masked volumes by applying a rigid image transformation using the image registration wizard tool in *Avizo*. The resulting volumes are cropped longitudinally by 50 voxels from both top and bottom to ensure uniform volumes. By using an anisotropic deformation to register either the wet to the dry image or vice versa, an optimal fit is achieved. The transformation parameters obtained from this process provide a quantitative measure of swelling and shrinkage through the scale factors corresponding to the inherent *x*, *y* and *z* axes.

## 3. Results and discussion

**Figure 3:**
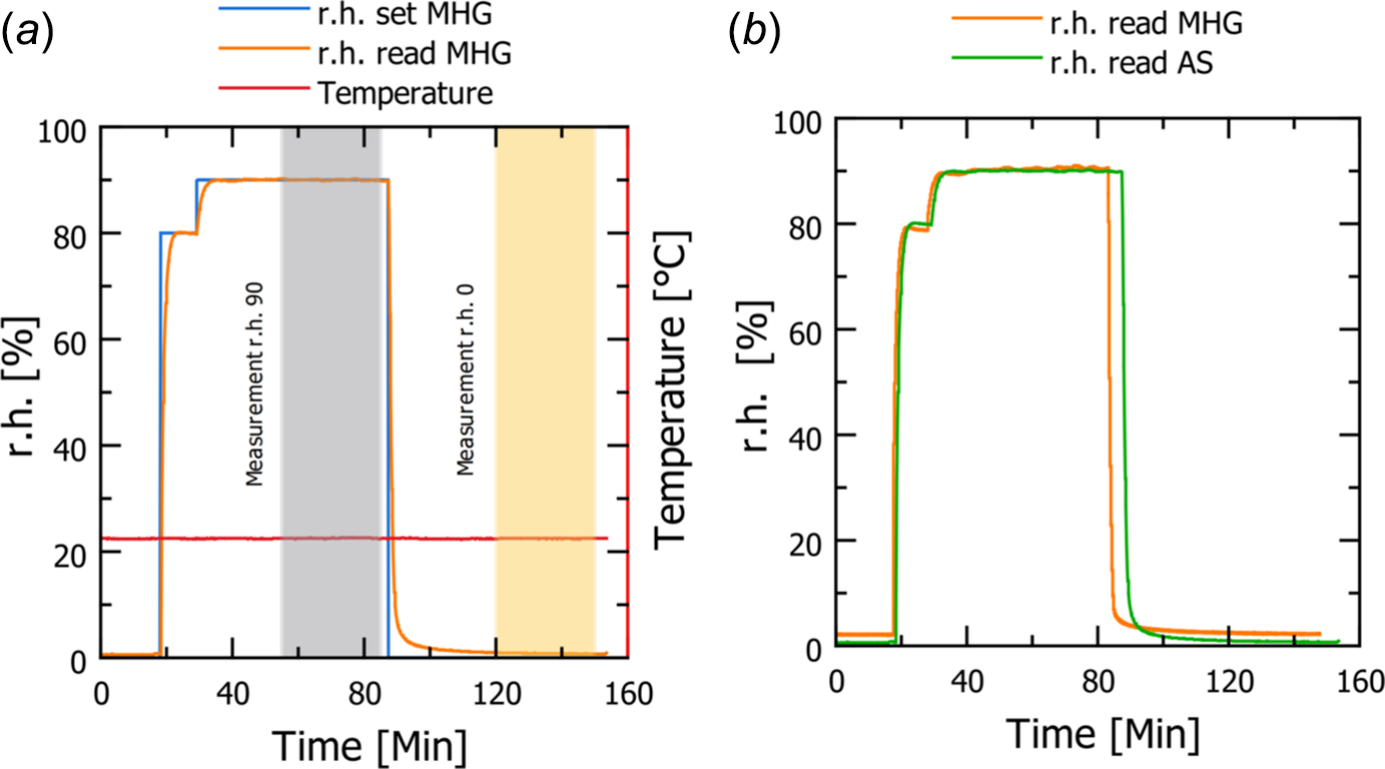
(*a*) Data for relative humidity and temperature in the inner chamber for absorption (90 ± 0.3% r.h.) and desorption (0 ± 0.2% r.h.) experiments. An intermediate step at 80% r.h. was necessary for reaching equilibrium at 90% r.h. faster and to avoid overshooting; grey and yellow vertical fields indicate the timepoints of the two presented experiments. (*b*) Recorded data are a comparison of r.h. between the MHG Sensor and an additional Arduino sensor (AS) over time. The time lag of the Arduino sensor is due to the difference in location within the climate chamber.

Conditioned air needs to be introduced into the climate chamber in a way that ensures that the biological sample is evenly conditioned in terms of time and space. In extensive tests, flow rates of 8 l min^−1^ seem to be an appropriate compromise between avoiding disturbance due to air flow and reaching equilibrium time as fast as possible. Further experiments revealed that a stable climate (r.h.) is reached after approximately 15 min (Fig. 3 ▸). Changing the climate conditions for a biological sample takes approximately an additional 30 min, depending on the thickness of the sample, in our case the cell wall. This has been validated by nanotomography subtracting images after the conditioning and after 30 min. Once the samples were successfully conditioned, there was no change detectable after subtraction of the corresponding tomographic images.

After the conditioning phase, samples are scanned in the holotomographic mode, which takes approximately 20 min per tomographic scan. Consequently, one experiment takes in total 1 h, including choosing the climate settings, sample conditioning and scanning of a sample. The ranges of possible stable climate conditions for the duration of an experiment are given in Table 1 ▸.

Monitoring the climate condition with two independent sensors [see Arduino sensor (2) and a modular humidity generator sensor (MHG, 4) in Fig. 1 ▸] during the experiments shows that both sensors collect similar climate data, which indicates stable and defined conditions (Fig. 3 ▸). In particular, the MHG sensor [(4) in Fig. 1 ▸] allows constant cross checking of the climate as it is the relevant sensor for controlling the humidity output. Heated sheathing of the MGH allows for stable r.h. at temperature levels, which can be deviating from room temperature, minimizing the risk of water condensation. However, condensation cannot be fully prevented, therefore we deliberately designed a transparent climate chamber [Fig. 1 ▸(*a*)] to ensure the visibility of water condensation. Condensation typically occurs at the saturation limits within the chamber, which limits our setup to a minimum temperature of 10°C at a relative humidity of 90% and to a relative humidity of 60% at 50°C. The climate chamber can be dried within minutes if condensation occurs through an opening at the bottom of the chamber [(7) and (8) in Fig. 1 ▸]. This opening allows moist air and condensation to be flushed out of the chamber by applying dry air at maximum flow rates (Table 1 ▸).

Compressed air was used for climatization since it allows for a high flow rate of the carrier gas. Hence, the lower limit of the relative humidity (r.h.) in the conditioned air (lowest r.h.) depends on the water content of the compressed air supply. Therefore, a target humidity of 0% r.h. was not feasible and 1% (± 0.2%) has been reached. On the other hand, the maximum r.h. of 90% with a stability of ± 0.3% has been achieved.

Saturated salt solutions are often used to adjust the humidity in climate chambers, with a wide range of r.h. (Greenspan, 1977 ▸). However, this approach is very time consuming if sets of different stable climate parameters are desired, requiring a frequent change of the salt solutions. Also, reaching target values above the level of a saturated salt solution is cumbersome. In addition, the salt solutions are highly corrosive, and they do cause degradation if accidentally spilled or leakage of the reservoir occurs. Therefore, we developed a climate chamber that is combined with a humidity generator which allows for a continuous air flow with defined and flexible adjustable r.h., avoiding the aforementioned issues. Another advantage of our setup is the continuous adjustability of the r.h. within the specified ranges (Table 1 ▸) when compared with saturated salt solutions. This approach therefore enables dynamic experiments during humidification thanks to the continuous adjustment of the humidity levels by using the humidity generator. Another advantage of using this humidity generator is the high flow rate of air. It reduces the risk of condensation, and it also allows samples to be analysed that specifically require dry conditions. This advantage is, for example, helpful for ZPC measurements of critical point dried organic samples, where an increase in r.h. can lead to movement artefacts. These artefacts can be minimized by keeping the r.h. level low and constant during the measurement.

**Figure 4:**
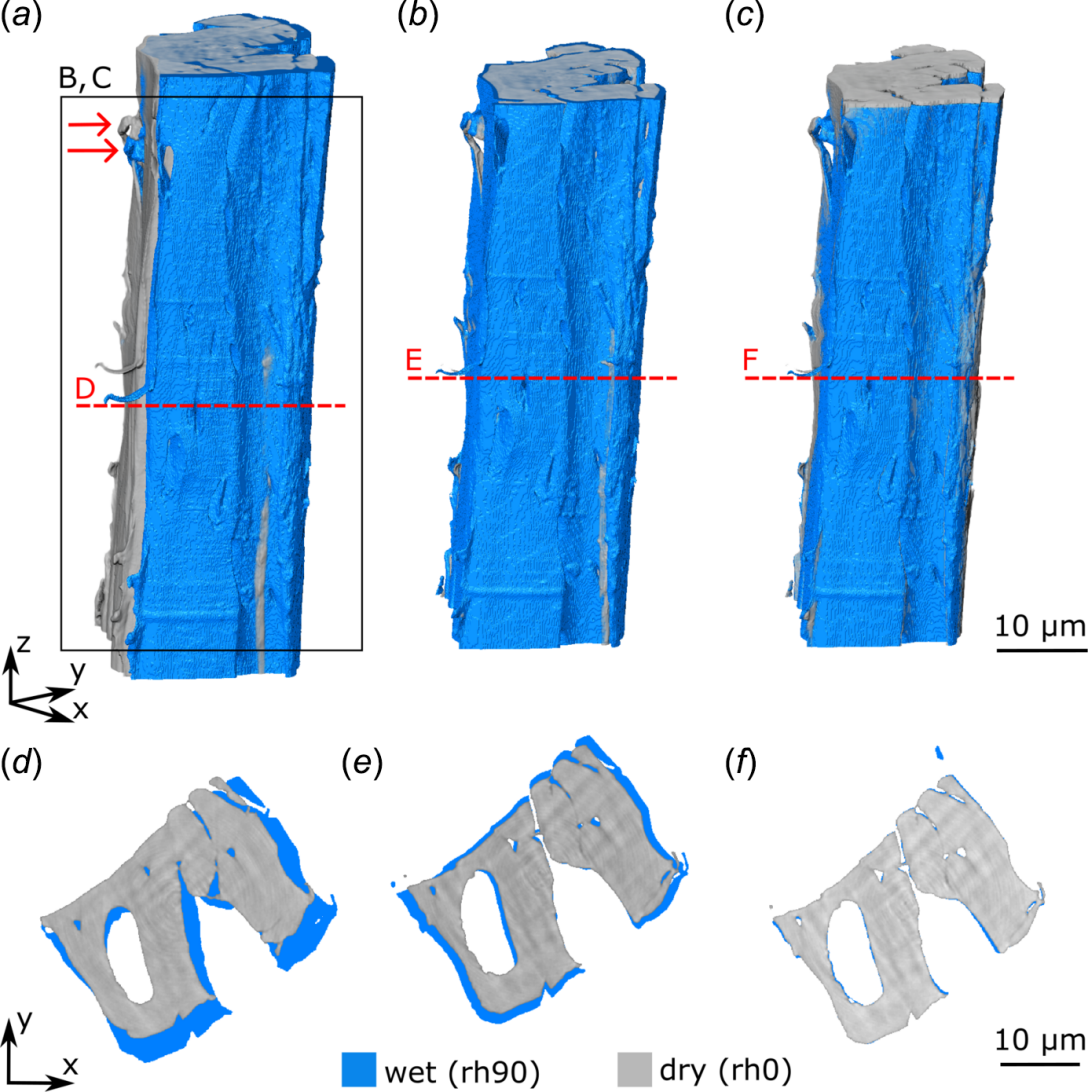
Volume rendering and cross section area of a nanotomographic scan of endocarp cells of *H. crepitans*. *In situ* nanotomography scans acquired at the beamline P05 in combination with the climate chamber. Blue depicts the cell wall at 90% relative humidity (r.h. 90) and grey is the same cell wall at 0% relative humidity (r.h. 0). (*a*) Volume rendering of the datasets obtained after reconstruction. (*b*) Volume rendering after rigid registration to align the volumes. To prevent protruding elements along the length (*z*-axis), the volume was cropped. (*c*) Volume rendering after affine registration to deform one dataset into the other to read out the scaling factors. (*d*) Cross sectional view of the unaligned datasets. (*e*) Cross sectional view of the rigidly aligned datasets. (*f*) Cross sectional view of the affinely registered datasets with small misalignments along the edges.

We tested the functionality of the climate chamber on an organic sample (lignified tissue of the endocarp of a *Hura crepitans* fruit) that has different material moisture levels depending on the ambient conditions (Fig. 4 ▸). Changing the climate from a dry (0% r.h.) to a humid climate (90% r.h.) led to a change in cell wall volume due to water uptake (absorption) into the cell wall. This approximated volumetric change was quantified using affine registration and the deformation of the entire volume was calculated as scale factors corresponding to the inherent *x*, *y* and *z* axes. From dry to wet state, the scale factors were found to be *x* ≃ 7.6%,*y* ≃ 7.5% and *z* ≃ 0.2%, resulting in a volumetric swelling of ∼15.9%.

Testing for equilibrium times for the moisture content of the sample (here, the cell wall in Fig. 4 ▸) is necessary for each new type of sample (species, tissue *etc*.). Therefore, preliminary tests are necessary to determine the duration until moisture equilibrium for each sample type by means of projection image comparison in the wet and dry state before the main experiments are conducted. In addition, changes in the samples due to high radiation dose were not found after visual assessment but it was not possible to draw definitive conclusions at the molecular level. Our assessment is therefore based on the sample’s response to moisture. Since the samples continued to swell and shrink consistently – even after repeated exposure – we assume that the irradiation under the applied measurement conditions did not alter the sample. However, this assumption should be re-evaluated for different samples and experimental conditions. Petruzzellis *et al.* (2018 ▸) detected cellular damage caused by synchrotron based microcomputed tomography but they inspected living cells and the damage was detected in cell membranes and RNA integrity.

Generally, the method is not limited to a specific specimen, since the tunable r.h. offers the possibility to measure factors such as (i) density differences in samples (*e.g.* in wood) induced by moisture content (Nopens *et al.*, 2019 ▸), (ii) flow analysis in small capillary systems (Martin *et al.*, 2022 ▸), (iii) interactions of biological organs like pits at different water pressure levels (Koddenberg *et al.*, 2021 ▸) and (iv) investigations of moisture induced movement within bulk sample material (Hill *et al.*, 2024 ▸). This allows for a functional analysis of biological samples. The setup is fully integrated into the beamline infrastructure. Therefore, it allows the temperature as well as the r.h. adjustment to be remotely controlled from the control hutch. Also, the data logging of the values (*T*, r.h.) is integrated. The samples can be examined at different climate conditions without having to re-enter the experimental hutch. Thanks to the climate cell it is now possible to analyse biological or other hygroscopic samples at stable climate conditions using the nanotomography setup at the P05 imaging beamline. All types of acquisition modes offered to users at the P05 nano instrument can be operated with the climate chamber. The easy and practical design allows this concept to also be transferred to other imaging beamlines.

## 4. Conclusions

Here, a newly designed and integrated climate chamber for analysing moisture sensitive samples at submicrometre resolution is presented. General requirements for a climate chamber as well as the special requirements of nanotomography are combined without compromising on data quality. The developed system allows the relative humidity and the temperature to be controlled and adjusted in an easy and practical way remotely from the control hutch. The climate conditions are stable over hours and therefore cover also longer tomography experiments. Care was taken to prevent any effect of the air flow on the stability of the sample during the experiment. The functionality of the climate chamber was thoroughly tested on the swelling and shrinkage behaviour of a biological sample. While condensation is always a problem when it comes to humidity control, it can be handled here thanks to the combination of the humidity generator and the transparent chamber. Long-term drift or vibrational motions are minimized due to the floating climate chamber design. A key feature for phase contrast nanotomography is the windowless approach, which avoids artefacts in the phase reconstruction caused by deposits on the X-ray passage. This setup is now available to all users at the nanotomography instrument at the imaging beamline P05 operated by Hereon, at the PETRA III storage ring at DESY. The practical and easy concept itself can easily be adapted to other nanotomography experiments at other beamlines worldwide.

## Figures and Tables

**Table 1:** Range of climate settings resulting in stable conditions in the chamber. Maximum and minimum values for the relative humidity (r.h. in %), temperature (°C) and flow rate (l min^−1^) are given

	Minimum	Maximum
r.h. (%)	0 ± 0.2%	90 ± 0.3%
Temperature (°C)	10	50 with r.h. 60% maximum
Flow rate (l min^−1^)	0	15
